# Mechanical properties of freely suspended semiconducting graphene-like layers based on MoS_2_

**DOI:** 10.1186/1556-276X-7-233

**Published:** 2012-04-25

**Authors:** Andres Castellanos-Gomez, Menno Poot, Gary A Steele, Herre SJ van der Zant, Nicolás Agraït, Gabino Rubio-Bollinger

**Affiliations:** 1 Kavli Institute of Nanoscience, Delft University of Technology, Lorentzweg 1, Delft 2628, CJ, The Netherlands; 2 Departamento de Física de la Materia Condensada (C-III), Universidad Autónoma de Madrid, Campus de Cantoblanco, Madrid E-28049, Spain; 3 Instituto Madrileño de Estudios Avanzados en Nanociencia (IMDEA-Nanociencia), Madrid E-28049, Spain; 4 Department of Engineering Science, Yale University, Becton 215, 15 Prospect St., New Haven, CT 06520, USA

**Keywords:** Molybdenum disulfide nanosheets, Freely suspended, Mechanical properties, Atomically thin crystal, Mechanical exfoliation

## Abstract

We fabricate freely suspended nanosheets of molybdenum disulphide (MoS_2_) which are characterized by quantitative optical microscopy and high-resolution friction force microscopy. We study the elastic deformation of freely suspended nanosheets of MoS_2 _using an atomic force microscope. The Young's modulus and the initial pre-tension of the nanosheets are determined by performing a nanoscopic version of a bending test experiment. MoS_2 _sheets show high elasticity and an extremely high Young's modulus (0.30 TPa, 50% larger than steel). These results make them a potential alternative to graphene in applications requiring flexible semiconductor materials.

PACS, 73.61.Le, other inorganic semiconductors, 68.65.Ac, multilayers, 62.20.de, elastic moduli, 81.40.Jj, elasticity and anelasticity, stress-strain relations.

## Background

The application of graphene in semiconducting devices is hindered by its lack of a bandgap. Up to now, two different strategies have been employed to fabricate semiconducting two-dimensional crystals: opening a bandgap in graphene [1-3] or using another two-dimensional crystal with a large intrinsic bandgap [4]. Atomically thin molybdenum disulphide (MoS_2_), a semiconducting transition metal dichalcogenide, has recently attracted a lot of attention due to its large intrinsic bandgap of 1.8 eV and high mobility μ > 200 cm^2 ^V^-1 ^s^-1 ^[5,6]. In fact, MoS_2 _has been employed to fabricate field-effect transistors with high on/off ratios [5], chemical sensors [7] and logic gates among other things [8]. Nevertheless, the study of the mechanical properties of this nanomaterial (which will dictate its applicability in flexible electronic applications) has just begun [9,10]. In a previous work, we studied the mechanical properties of freely suspended MoS_2 _nanosheets using a bending test experiment performed with the tip of an atomic force microscope (AFM) [10].

Here, we perform a more detailed characterization of the fabricated nanosheets by quantitative optical microscopy and high-resolution friction force microscopy, and we extend the study of the mechanical properties to a larger number of MoS_2 _nanosheets (with thicknesses in the range of 5 to 25 layers) to improve the robustness of our statistical analysis. We present force versus deformation curves measured not only by pushing the nanosheets (as usual) but also by pulling them, demonstrating that for moderate deformations pushing and pulling the nanosheets are equivalent. These measurements allow for the simultaneous determination of the Young's modulus (*E*) and the initial pre-tension (*T*) of these MoS_2 _nanosheets.

## Methods

Although atomically thin MoS_2 _crystals can be fabricated by scotch-tape-based micromechanical cleavage [11], this procedure can leave traces of adhesive. Thus, it is preferable to use an all-dry technique based on poly (dimethyl)-siloxane stamps which have been successfully employed to fabricate ultra-clean atomically thin crystals of graphene [12], graphene nanoribbons [13], NbSe_2_, MoS_2 _[14], and muscovite mica [15]. In order to fabricate freely suspended atomically thin MoS_2 _flakes, the cleaved flakes are transferred to a pre-patterned oxidized silicon wafer [16] with circular holes 1.1 μm in diameter and 200-nm deep.

**Figure 1:**
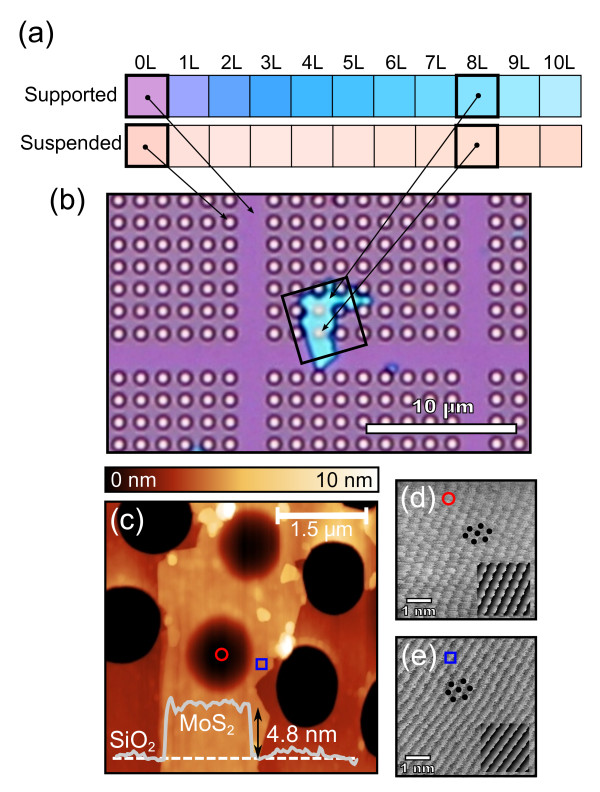
**Identification and characterization of freely suspended MoS_2 _nanosheets**. (**a**) Color chart displaying the calculated color for MoS_2 _nanosheets with different number of layers laying on the substrate (supported) or covering a hole (suspended). (**b**) Optical micrograph of a 4.8-nm-thick (8 layers) MoS_2 _flake deposited on top of a 285-nm SiO_2_/Si substrate pre-patterned with an array of holes 1.1 μm in diameter. Even though the flake covers two holes, it is thin (and transparent) enough to permit optical identification of the covered holes, which present a slightly different color from the uncovered holes, as predicted by the color chart shown in (a). (**c**) Contact mode AFM topography of the region marked by the rectangle in (b); (inset) topographic line profile acquired along the dashed line in (c). (**d**) and (**e**) Raw friction forward images acquired in contact mode AFM in a suspended and a supported region, marked by a red circle and a blue square in (c), respectively. The insets in (d) and (e) show two friction images simulated with a two-dimensional Tomlinson model. Both images have been simulated employing the same crystal lattice and orientation but different depth of the potential well (see text for a full discussion).

After fabrication, an optical microscope (Nikon eclipse LV100, Nikon Instruments Inc., Melville, NY, USA) is used to identify MoS_2 _flakes at first glance. In fact, ultrathin MoS_2 _flakes deposited onto a silicon wafer with a 285-nm-thick SiO_2 _capping layer can be easily identified by optical microscopy. Figure 1a shows a chart of the expected color of MoS_2 _flakes with different thicknesses when they are laying on the surface or covering a hole. The expected color has been calculated with a Fresnel law-based model, employing the refractive index of MoS_2 _in [14] and the response of the camera as indicated in [17]. The topography of selected flakes is then studied by contact mode atomic force microscopy to avoid possible artifacts in the flake thickness measurements [18]. Figure 1b, c is an optical micrograph and a contact mode AFM topography, respectively, of an 8-layer-thick MoS_2 _flake deposited onto a 285-nm SiO_2_/Si pre-patterned substrate.

Additionally, high resolution contact mode AFM measurements can provide lattice resolution even in the suspended region of the MoS_2 _flakes which demonstrates the very clean nature of our fabrication technique. Figure 1d, e shows two lateral force maps (friction images) obtained in the suspended and the supported parts of the MoS_2 _flake shown in Figure 1c. The atomic resolution can be better resolved in the suspended region of the MoS_2 _flake (Figure 1d), while in the supported part, the frictional force image mainly follows parallel stripes (Figure 1e). We have employed a two-dimensional Tomlinson model [19] to simulate the frictional force image measured in the supported part of the nanolayer (see inset in Figure 1e), finding a remarkable qualitative agreement. Interestingly, by reducing a 25% in the depth of the surface potential employed in the simulation the calculated friction force image qualitatively matches the one measured in the suspended part of the MoS_2 _nanomembrane (Figure 1d). This difference in the frictional force image can be due to a slight modification of the MoS_2 _lattice induced by the pre-tension of the suspended part of the sheet. However, a detailed analysis of the tension dependence of frictional force images and their interpretation, although interesting, is beyond the scope of this work.

## Results and discussion

**Figure 2:**
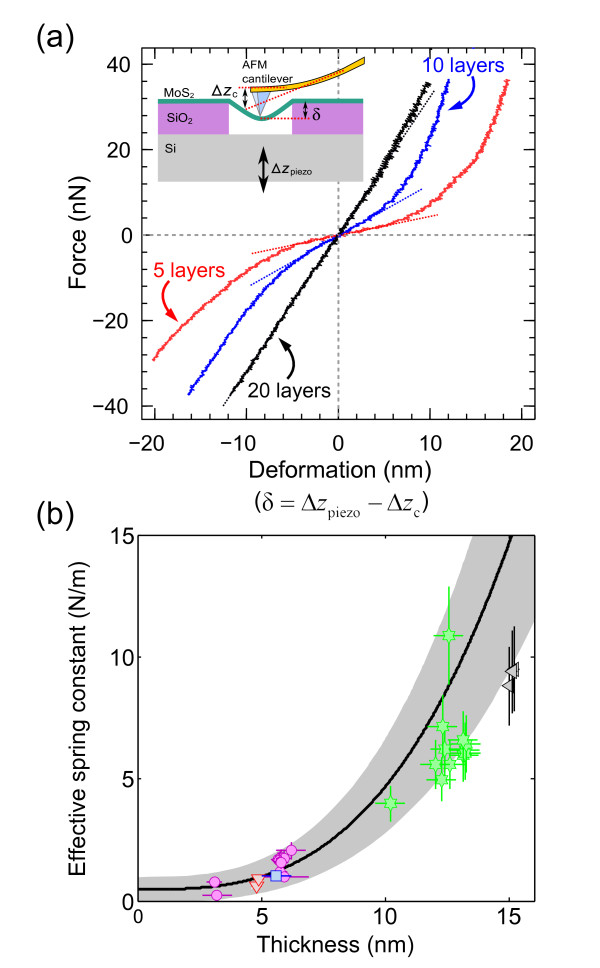
**Bending test experiment on suspended ultrathin MoS_2 _sheets**. (**a**) Force versus deformation traces measured by pushing and pulling at the center of the suspended part of MoS_2 _nanosheets with 5, 10, and 20 layers in thickness. The slope of the traces around zero deformation is marked by a dotted line. (**b**) Effective spring constant as a function of the thickness measured for 31 MoS_2 _suspended nanosheets with thickness ranging from 25 down to 5 layers. Data points sharing color and symbol correspond to suspended nanosheets from the same MoS_2 _flake. The solid black line shows the calculated relationship with Equation 2 using *E *= 0.30 TPa and *T *= 0.15 N/m. The gray area around the solid black line indicates the uncertainty of *E *and *T*: Δ*E *= ± 0.10 TPa and Δ*T *= ± 0.15 N/m.

Once the suspended nanosheet under study is identified and characterized, we measure its elastic mechanical properties using the AFM tip to apply a load cycle in the center of the suspended region of the nanosheet while its deformation is measured, as shown in the inset of Figure 2a. When the tip and sample are in contact, the elastic deformation of the nanosheet (*δ*), the deflection of the AFM cantilever (Δ*z*_c_), and the displacement of the scanning piezotube of the AFM (Δ*z*_piezo_) are related by the following equation:

(1) $$\delta =\Delta z_{\text{piezo}}-\Delta z_{\text{c}}$$

The force applied is related to the cantilever deflection as *F *= *k*_c_·Δ*z*_c_, where *k*_c _is the spring constant of the cantilever (*k*_c _= 0.75 ± 0.20 N/m [20]).

Figure 2a shows three different deformations versus force (*F*(*δ*) hereafter), measured for nanosheets with 5, 10, and 20 layers in thickness, not only by pushing the sheets but also by pulling them. For small deformations, these *F*(*δ*) traces are linear with a slope that defines the effective spring constant of the nanolayer (*k*_eff_) [21]:

(2) $$k_{\text{eff}}={\left(\frac{\partial F}{\partial \delta }\right|}_{\delta =0}=\frac{4\pi E}{3\left(1-\nu^{2}\right)}\cdot \left(\frac{t^{3}}{R^{2}}\right)+\pi T$$

with *ν *the Poisson's ratio (*ν *= 0.125, [22]), *t *the thickness, and *R *the radius of the nanosheet. As the effective spring constant depends on both the Young's modulus and the pre-tension constant, one cannot separately determine these values just from the slope of a *F*(*δ*) trace. To independently determine *E *and *T*, however, one can use the thickness dependence of the effective spring constant. Indeed, according to Equation 2, the first term (which accounts for the bending rigidity of the layer) strongly depends on the sheet thickness, while the second one (which accounts for the initial pre-tension) is thickness independent. Fitting the measured *k*_eff _versus thickness to Equation 2, one can determine *E *and *T*. Figure 2b shows the measured *k*_eff _as a function of the thickness of 31 different MoS_2 _layers and the fit to the experimental data using the following:

(3) E=0.30±0.10 TPa andT=0.15±0.15 N/m

This Young's modulus value is extremely high, only one third lower than exfoliated graphene (one of the stiffest materials on earth with *E *= 0.8 to 1.0 TPa) [23,24] and comparable to other 2D crystals such as graphene oxide (0.2 TPa) [25] or hexagonal boron nitride (0.25 TPa) [26]. It is also remarkable that the *E *value is restrained between 0.2 and 0.4 TPa, indicating a high homogeneity of the MoS_2 _flakes, which is much smaller than the one observed for graphene (0.02 to 3 TPa) [27] or graphene oxide (0.08 to 0.7 TPa) [25]. The high Young's modulus of the ultrathin MoS_2 _flakes (*E *= 0.30 ± 0.10 TPa compared to the bulk value *E*_bulk _= 0.24 TPa [28]) can be explained by a low presence of stacking faults. Indeed, the thinner the nanosheet the lower the presence of stacking faults, allowing the study of the intrinsic mechanical properties of the material.

## Conclusion

We have studied the mechanical properties of ultrathin freely suspended MoS_2 _nanosheets with 5 to 25 layers thick. The mean Young's modulus of these suspended nanosheets, *E *= 0.30 ± 0.07 TPa, is extremely high, and they present low pre-strain and high strength, being able to stand elastic deformations of tens of nanometers elastically without breaking. In summary, the low pre-tension and high elasticity and Young's modulus of these crystals make them attractive substitutes or alternatives for graphene in applications requiring flexible semiconductor materials.

## Abbreviations

AFM: Atomic force microscope; MoS_2_: Molybdenum disulphide.

## Competing interests

The authors declare that they have no competing interests.

## Authors' contributions

AC-G carried out the transfer and characterization of MoS_2 _nanolayers and the bending test measurements. MP fabricated the pre-patterned substrates. AC-G and GR-B participated in the design and coordination of the experiments and designed the manuscript layout. MP, GAS, HSJvdZ, and NA participated in the drafting of the manuscript and helped with the interpretation of the data. All authors read and approved the final manuscript.

## Authors' information

AC-G and MP are post-doctoral researchers at the Kavli Institute of Nanoscience at Delft University of Technology and at the Department of Engineering Science of Yale University, respectively. GAS and HSJvdZ are assistant professor and full professor, respectively, at the Kavli Institute of Nanoscience at Delft University of Technology. NA and GR-B are associate professor and full professor, respectively, at the Department of Condensed Matter at Universidad Autonoma de Madrid. NA is also an associated senior researcher at the Madrid Institute for Advanced Studies in Nanoscience (IMDEA-Nanoscience).

## References

[B1] Chen Z Lin Y Rooks M Avouris P Graphene nano-ribbon electronics Physica E 2007 40 228 232 10.1016/j.physe.2007.06.020

[B2] Li X Wang X Zhang L Lee S Dai H Chemically derived, ultrasmooth graphene nanoribbon semiconductors Science 2008 319 1229 10.1126/science.1150878 18218865

[B3] Castellanos-Gomez A Wojtaszek M Tombros N van Wees BJ Reversible hydrogenation and bandgap opening of graphene and graphite surfaces probed by scanning tunneling spectroscopy Small 2012 8 1607 1613 10.1002/smll.201101908 22431189

[B4] Podzorov V Gershenson ME Kloc C Zeis R Bucher E High-mobility field-effect transistors based on transition metal dichalcogenides Appl Phys Lett 2004 84 3301 10.1063/1.1723695

[B5] Radisavljevic B Radenovic A Brivio J Giacometti V Kis A Single-layer MoS_2 _transistors Nat Nanotechnol 2011 6 147 150 21278752 10.1038/nnano.2010.279

[B6] Korn T Heydrich S Hirmer M Schmutzler J Schüller C Low-temperature photocarrier dynamics in monolayer MoS_2_ Appl Phys Lett 2011 99 102109 10.1063/1.3636402

[B7] Li H Yin Z He Q Huang X Lu G Fam DWH Tok AIY Zhang Q Zhang H Fabrication of single- and multilayer MoS_2 _film-based field-effect transistors for sensing NO at room remperature Small 2012 8 63 67 10.1002/smll.201101016 22012880

[B8] Radisavljevic B Whitwick MB Kis A Integrated circuits and logic operations based on single-layer MoS_2_ ACS Nano 2011 5 9934 9938 10.1021/nn203715c 22073905

[B9] Bertolazzi S Brivio J Kis A Stretching and breaking of ultrathin MoS_2_ ACS Nano 2011 5 9703 9709 10.1021/nn203879f 22087740

[B10] Castellanos-Gomez A Poot M Steele GA Van der Zant HSJ Agraït N Rubio-Bollinger G Elastic properties of freely suspended MoS_2 _nanosheets Adv Mater 2012 24 772 775 10.1002/adma.201103965 22231284

[B11] Novoselov K Jiang D Schedin F Booth T Khotkevich V Morozov S Geim A Two-dimensional atomic crystals Proc Natl Acad Sci USA 2005 102 10451 10.1073/pnas.0502848102 16027370 PMC1180777

[B12] Castellanos-Gomez A Smit RHM Agraït N Rubio-Bollinger G Spatially resolved electronic inhomogeneities of graphene due to subsurface charges Carbon 2012 50 932 938 10.1016/j.carbon.2011.09.055

[B13] Moreno-Moreno M Castellanos-Gomez A Rubio-Bollinger G Gomez-Herrero J Agraït N Ultralong natural graphene nanoribbons and their electrical conductivity Small 2009 5 924 927 10.1002/smll.200801442 19242945

[B14] Castellanos-Gomez A Agrait N Rubio-Bollinger G Optical identification of atomically thin dichalcogenide crystals Appl Phys Lett 2010 96 213116 10.1063/1.3442495

[B15] Castellanos-Gomez A Wojtaszek M Tombros N Agraït N van Wees BJ Rubio-Bollinger G Atomically thin mica flakes and their application as ultrathin insulating substrates for graphene Small 2011 7 2491 2497 10.1002/smll.201100733 21805626

[B16] Witkamp B Poot M van der Zant H Bending-mode vibration of a suspended nanotube resonator Nano Lett 2006 6 2904 2908 10.1021/nl062206p 17163728

[B17] Henrie J Kellis S Schultz S Hawkins A Electronic color charts for dielectric films on silicon Opt Express 2004 12 1464 1469 10.1364/OPEX.12.001464 19474970

[B18] Nemes-Incze P Osváth Z Kamarás K Biró LP Anomalies in thickness measurements of graphene and few layer graphite crystals by tapping mode atomic force microscopy Carbon 2008 46 1435 1442 10.1016/j.carbon.2008.06.022

[B19] Sasaki N Kobayashi K Tsukada M Atomic-scale friction image of graphite in atomic-force microscopy Phys Rev B 1996 54 2138 10.1103/PhysRevB.54.2138 9986064

[B20] Sader JE Chon JWM Mulvaney P Calibration of rectangular atomic force microscope cantilevers Rev Sci Instrum 1999 70 3967 10.1063/1.1150021

[B21] Landau L Lifshitz E Theory of Elasticity 1959 3 Stoneham: Butterworth-Heinemann

[B22] Lovell M Khonsari M Marangoni R A finite element analysis of the frictional forces between a cylindrical bearing element and MoS_2 _coated and uncoated surfaces Wear 1996 194 60 70 10.1016/0043-1648(95)06708-6

[B23] Lee C Wei X Kysar JW Hone J Measurement of the elastic properties and intrinsic strength of monolayer graphene Science 2008 321 385 10.1126/science.1157996 18635798

[B24] Li P You Z Haugstad G Cui T Graphene fixed-end beam arrays based on mechanical exfoliation Appl Phys Lett 2011 98 253105 10.1063/1.3594242

[B25] Gómez-Navarro C Burghard M Kern K Elastic properties of chemically derived single graphene sheets Nano Lett 2008 8 2045 2049 10.1021/nl801384y 18540659

[B26] Song L Ci L Lu H Sorokin PB Jin C Ni J Kvashnin AG Kvashnin DG Lou J Yakobson BI Ajayan PM Large scale growth and characterization of atomic hexagonal boron nitride layers Nano Lett 2010 10 3209 3215 10.1021/nl1022139 20698639

[B27] Poot M Van der Zant HSJ Nanomechanical properties of few-layer graphene membranes Appl Phys Lett 2008 92 063111

[B28] Feldman J Elastic constants of 2 H-MoS_2 _and 2 H-NbSe_2 _extracted from measured dispersion curves and linear compressibilities J Phys Chem Solids 1976 37 1141 1144 10.1016/0022-3697(76)90143-8

